# PRE-ECV Score: Validation of a Pre-Specified Tool for Predicting the Success of External Cephalic Version at Term—Proof of Concept

**DOI:** 10.3390/jcm15020724

**Published:** 2026-01-15

**Authors:** Maisa Manasar-Dyrbuś, Kacper Niziński, Kaja Skowronek, Ewa Winkowska, Rafał Stojko, Jakub Staniczek

**Affiliations:** Department of Gynecology, Obstetrics and Gynecological Oncology, Medical University of Silesia, 40-211 Katowice, Poland

**Keywords:** external cephalic version, breech presentation, ECV

## Abstract

**Objectives:** We aimed to validate the predictive performance of the PRE-ECV Score, a pre-specified clinical tool, using logistic recalibration and bootstrap optimism correction to estimate the probability of successful external cephalic version (ECV) at term. **Methods:** The PRE-ECV Score was defined *a priori* based on a literature review and expert consensus, incorporating eight variables supported by level A or B scientific evidence (total score range 0–13). Validation of this pre-specified score was performed in a retrospective, single-center cohort of 100 consecutive ECV procedures between November 2023 and October 2025. Study conduct and reporting adhered to the Transparent Reporting of a multivariable prediction model for Individual Prognosis or Diagnosis (TRIPOD) statement. **Results:** The revised PRE-ECV Score demonstrated moderate discrimination (AUC = 0.76; 95% CI, 0.66–0.85). Calibration was moderate, with a bootstrap-corrected intercept of −3.02 (95% CI, −5.42 to −1.51) and a calibration slope of 0.70 (95% CI, 0.44–1.18). Observed ECV success rates increased across score strata, from 34.6% (0–4 points) to 79.4% (5–8 points) and 100% (≥9 points). **Conclusions:** The PRE-ECV Score demonstrated moderate discrimination and satisfactory calibration in this single-center validation. Given the small sample and model optimism, these results should be interpreted strictly as proof-of-concept.

## 1. Introduction

Breech presentation at term complicates approximately 3–4% of singleton pregnancies and is strongly associated with an increased risk of cesarean delivery [1]. Cesarean section rates have risen dramatically worldwide, with recent estimates approaching 50% in several regions, including Poland [2,3]. Reducing unnecessary cesarean deliveries has become a global public health priority [1]. External cephalic version (ECV) is a safe and effective intervention recommended by international guidelines to reduce the incidence of breech presentation and thereby lower cesarean section rates. According to the Royal College of Obstetricians and Gynaecologists Green-top Guideline No. 20a, ECV should be routinely offered at term, as supported also by the Cochrane review [4,5].

Success rates of ECV vary widely between 35% and 86%, influenced by maternal, fetal, and procedural factors [1,2,6]. Multiparity, non-engagement of the breech, a palpable fetal head, posterior placental location, adequate amniotic fluid volume, and the use of tocolysis have all been shown to strongly increase the likelihood of success [7,8,9,10]. While numerous predictors of ECV success have been described, multivariable tools and clinical scores proposed to combine them have generally shown modest discrimination and inconsistent calibration [1,11,12,13,14,15].

Despite accumulating evidence, the implementation of ECV remains inconsistent, particularly in countries with high cesarean section rates such as Poland. Recent national surveys confirm low awareness and uptake among obstetricians and midwives, despite evidence of safety and effectiveness [3,16]. There is, therefore, a need for a simple, reliable, and clinically applicable scoring system to support decision-making and counseling.

To address this gap, we designed the PRE-ECV Score, a pragmatic tool based on eight clinical and ultrasonographic variables supported by level A and B evidence. The present study aimed to validate this pre-specified score in a consecutive cohort of women undergoing ECV at term. We assessed its predictive performance through logistic recalibration and applied bootstrap resampling to obtain optimism-corrected estimates, in line with TRIPOD recommendations.

## 2. Methods

### 2.1. Study Design and Setting

The PRE-ECV Score was defined based on a literature review and expert consensus. In this study, we conducted a single-center, retrospective validation of this pre-specified score in a cohort of women undergoing ECV procedures at the Clinical Department of Gynecology, Obstetrics, and Gynecological Oncology, Medical University of Silesia in Katowice (Markiefki 87, 40-211 Katowice, Poland) between November 2023 and October 2025. Performance was evaluated through logistic recalibration (intercept and slope) and bootstrap resampling to correct for optimism, in line with TRIPOD recommendations [17]. Records from 100 consecutive ECV procedures in singleton term pregnancies (gestational age ≥ 37 weeks) with breech or transverse presentation were analyzed. The primary outcome, successful ECV, was defined as immediate conversion of the fetus to cephalic presentation confirmed by ultrasound at the end of the procedure. All women undergoing ECV during the study period were included; no cases were excluded.

### 2.2. Predictor Variables and Scoring System

The PRE-ECV Score was developed *a priori* based on a literature review [1,2,3,4,5,6,7,8,9,10] and expert consensus among all of the authors. The total score ranges from 0 to 13 points and incorporates eight clinical and ultrasonographic variables supported by level A (2 points) or B (1 point) scientific evidence. All predictors were assessed immediately prior to the ECV procedure by the attending obstetrician. ECV success was determined by ultrasound at the end of the procedure. Outcome assessors were not blinded to predictor information. The PRE-ECV Score is presented in Table 1.

### 2.3. Statistical Analysis

Candidate predictors were combined into an additive point score ranging from 0 to 13. The scoring system was fully prespecified before outcome evaluation, with no data-driven variable selection or coefficient optimization. To map the pre-specified score to predicted probabilities between the total score and the probability of successful ECV, we fitted a logistic regression model with the total score as the sole predictor, thereby ensuring model simplicity and avoiding overfitting.

Model discrimination was quantified using the area under the receiver-operating characteristic curve (AUC). Calibration was assessed visually using a calibration plot with a locally weighted regression (LOESS-smoothed) curve overlaid on the ideal 45° reference line.

Given the modest sample size (N = 100) and anticipated optimism, we performed bootstrap optimism correction for model performance metrics using 2000 bootstrap resamples. In each bootstrap sample, the model was refitted, and performance was evaluated both within the bootstrap sample and in the original dataset to estimate optimism. Optimism-corrected AUC, calibration intercept, and calibration slope are reported alongside apparent values.

For clinical interpretability, we calculated observed ECV success rates (with 95% Wilson confidence intervals) across three prespecified strata (0–4, 5–8, and ≥9 points). There were no missing data for predictors or outcome. This was a consecutive sample cohort, and no a priori sample size calculation was performed. All analyses were conducted using Python version 3.11 (Python Software Foundation, Wilmington, DE, USA).

## 3. Results

### 3.1. Study Population

Among 100 ECV attempts, 69 were successful, yielding an overall success rate of 69.0%. The revised PRE-ECV Score was significantly higher in the successful group (median = 6; IQR 5–7) than in the unsuccessful group (median = 4; IQR 4–6; *p* < 0.001, Mann–Whitney U test). Baseline characteristics of the study population stratified by ECV outcome are summarized in Table 2

### 3.2. Discrimination, Calibration and Decision Curve Analysis

**Figure 1:**
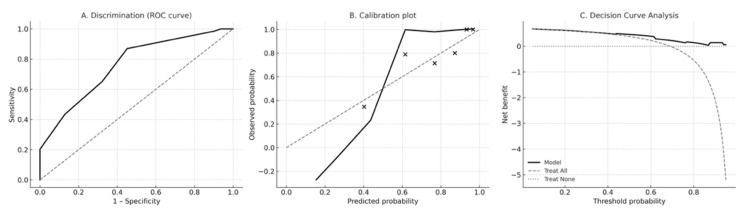
Model performance of the revised PRE-ECV Score. (**A**) Receiver-operating characteristic (ROC) curve demonstrating discrimination of the PRE-ECV Score for predicting successful external cephalic version (ECV). The apparent area under the ROC curve (AUC) was 0.76 with a bootstrap-derived 95% confidence interval of 0.66–0.85 (DeLong method). (**B**) Calibration plot showing agreement between predicted and observed probabilities of ECV success. The LOESS-smoothed calibration curve (solid black line) demonstrates moderate calibration relative to the ideal 45° reference line (gray dashed), consistent with a bootstrap-corrected calibration intercept of −3.02 and calibration slope of 0.70. Black points represent mean predicted and observed probabilities within deciles of predicted risk. (**C**) Decision curve analysis (DCA) evaluating the net clinical benefit of using the PRE-ECV Score across a range of threshold probabilities. The PRE-ECV model (solid black line) provides greater net benefit than “treat all” (gray dashed line) and “treat none” (gray dotted line) strategies for threshold probabilities between approximately 0.25 and 0.70, indicating meaningful potential for guiding clinical decision-making.

The revised PRE-ECV Score demonstrated moderate discrimination, with an apparent AUC of 0.76; (95% CI of 0.66–0.85; DeLong method). Bootstrap optimism-corrected calibration yielded an intercept of −3.02 (95% CI −5.42 to −1.51) and a calibration slope of 0.70 (95% CI 0.44–1.18), indicating moderate agreement between predicted and observed probabilities. Decision curve analysis showed that the model provided greater net clinical benefit than “treat all” or “treat none” strategies across a clinically relevant range of threshold probabilities (Figure 1).

### 3.3. Validation Sample Only

The Youden index identified a cutoff of ≥9 points in this cohort. The predicted probability of ECV success for a score of 9 was 0.97 in the apparent logistic model and 0.96 after bootstrap correction. These estimates reflect the prevalence and score distribution of this single-center sample and may be optimistic; therefore, threshold determination should be re-evaluated in external validation.

### 3.4. Clinical Utility

Decision-curve analysis (DCA) demonstrated that the PRE-ECV Score provided greater net benefit than either a “treat-all” or “treat-none” strategy across clinically relevant threshold probabilities ranging from 0.30 to 0.80. At the predefined cutoff of ≥9 points, the score achieved a sensitivity of 64% (95% CI, 52–75), a specificity of 80.6%, a positive predictive value of 88% (95% CI, 75–95), and a negative predictive value of 50% (95% CI, 38–62). These findings indicate balanced discriminatory performance and potential clinical utility, although threshold-based decision-making should be re-evaluated in future external validation studies.

## 4. Discussion

The PRE-ECV Score demonstrated moderate but clinically meaningful discrimination in predicting external cephalic version success, with an AUC of 0.76 (95% CI 0.66–0.85). Decision-curve analysis suggested a net clinical benefit of using the score, supporting its potential role as a decision aid. At the predefined cutoff of ≥9 points, the model achieved balanced diagnostic performance, with a clear separation between strata. These findings, although encouraging, should be interpreted as exploratory and hypothesis-generating, pending external validation.

Most previously proposed ECV prediction tools report mixed discrimination and variable calibration. In the widely cited Dahl et al. (2021) model (BMI, parity, placental location, presentation), the AUC was 0.667 (95% CI 0.634–0.701) with good calibration; the cohort’s overall ECV success rate was 40.6% [1]. External validation by Kishkovich et al. (2023) in an independent US cohort yielded AUC 0.70 (95% CI 0.65–0.75) with a 44.4% success rate, importantly, practice patterns differed substantially (neuraxial anesthesia 83.5% in the derivation setting vs. 10.4% in the validation cohort), underscoring spectrum/practice-mix effects on model transportability [11]. Earlier tools performed similarly or worse. De Hundt et al. (2012) externally validated a prior model and found AUC 0.66 (95% CI 0.60–0.72) with systematic underestimation of success by 4–14%, indicating miscalibration in a new setting [12]. The clinical score by Burgos et al. (2012) reported a predictive capacity 70.1% (95% CI 66.9–73.4) [14]. The GNK-PIMS model proposed by Tasnim et al. has shown the weakest performance among commonly cited tools in comparative work [15]. Recent head-to-head testing by Yerrabelli et al. [13] (2025)—evaluating six models in a single US institution—found Dahl et al. [1] (2021) to be the best performer (AUC 0.779; bootstrapped 95% CI 0.71–0.84) and well-calibrated, whereas Tasnim et al. [15] (2012) performed worst (AUC 0.626) and others clustered around AUC 0.68–0.71.

The strengths of this study include the use of prespecified predictors derived from prior literature and expert consensus; a comprehensive evaluation of model performance encompassing discrimination, multiple calibration indices, and decision curve analysis; and the development of a transparent, point-based scoring system that has the potential to be simple to apply in clinical practice if validated externally.

Limitations relate to its retrospective, single-center design and modest sample size (N = 100), which raise concerns about spectrum effects and residual confounding. The AUC observed in our cohort likely reflects residual optimism due to the sample size and relatively high number of prespecified predictors. Although bootstrap correction partially accounts for optimism, it remains a form of “testing on ourselves” and cannot guarantee reproducibility in independent data. Accordingly, PRE-ECV should be regarded solely as a proof-of-concept model. While the high apparent AUC reflects potential, it also highlights the risk of overfitting in a small, single-center dataset. Only external, multicenter validation will determine whether the model has reproducible value, and until then, no clinical implementation should be attempted.

Moreover, we emphasize that a successful external cephalic version (ECV) does not automatically result in cephalic presentation at delivery or vaginal birth. However, immediate ECV success is the most direct and procedure-specific outcome and can be clearly attributed to the predictors included in the score. Evaluating downstream outcomes, such as mode of delivery, would introduce post-procedural factors and practice-related variability; therefore, this was deliberately avoided in this proof-of-concept validation.

Further research should prioritize multicenter validation and potential recalibration. In addition, cost-effectiveness analyses, such as those conducted by Tan et al., may help clarify whether targeted use of adjuncts in moderate-probability groups is economically and clinically advantageous [18]. Finally, compared with published cohorts—Dahl (2021) [1] (success 40.6%), Kishkovich (2023) [11] (44.4%), and Yerrabelli (2025) [13] (52.2%)—our center’s success rate (69%) was higher, which may inflates apparent AUC and net benefit. Differences in operator experience, patient selection, and anesthesia/tocolysis protocols likely contribute and should be considered when applying PRE-ECV outside our setting. External validation in centers with diverse practice patterns will be necessary to confirm calibration and net benefit.

## 5. Conclusions

The PRE-ECV Score represents a proof-of-concept model that demonstrated apparently strong performance within a small, single-center validation cohort. Its relatively high apparent discrimination likely reflects sample-specific characteristics and residual overfitting rather than true generalizable accuracy. These findings should therefore be interpreted as preliminary evidence of potential utility rather than as a clinically deployable tool. External, multicenter validation is essential before considering any clinical implementation.

## Figures and Tables

**Table 1:** PRE-ECV Score.

Factor	Condition	Points
**Parity**	Multiparous	2
	Nulliparous	0
**Fetal presentation**	Transverse	2
	Complete breech	1
	Frank breech	0
**Non-engagement of the presenting part**	Yes (presenting part not engaged)	2
	No (presenting part engaged)	0
**Palpable fetal head**	Yes	2
	No	0
**Estimated fetal weight (EFW)**	≥10th percentile	1
	≤9th percentile	0
**Maximum vertical pocket (MVP)**	≥4 cm	1
	≤3 cm	0
**Placental location**	Posterior wall	1
	Anterior wall	0
**Tocolysis prior to ECV**	Yes	2
	No	0

**Table 2:** Characteristics of the study population stratified by ECV, stratified according to procedural outcome. Values are presented as median (IQR) or N (%). Percentages are calculated per column.

Characteristic	All Cohort (N = 100)	Successful ECV (n = 69)	Unsuccessful ECV (n = 31)	*p*-Value
**Maternal age** **(years)**	31 (IQR 29–33)	31 (IQR 29–33)	31 (IQR 28.5–33)	0.81
**Gestational age at ECV** **(weeks)**	38 (IQR 37–38)	38 (IQR 37–38)	38 (IQR 37–38)	0.90
**Parity**				0.087
Multiparous	55 (55.0%)	42 (60.9%)	13 (41.9%)	
Nulliparous	45 (45.0%)	27 (39.1%)	18 (58.1%)	
**Fetal presentation**				
Transverse	8 (8.0%)	7 (10.1%)	1 (3.2%)	0.14
Complete breech	16 (16.0%)	11 (15.9%)	5 (16.1%)	
Frank breech	76 (76.0%)	50 (72.5%)	26 (83.9%)	
**Non-engagement of presenting part**	36 (36.0%)	7 (10.1%)	29 (93.5%)	<0.001
**Palpable fetal head**	69 (63.0%)	63 (91.3%)	6 (8.7%)	<0.001
**Estimated fetal weight (EFW)**				1.00
≤9th percentile	20 (20.0%)	14 (20.3%)	6 (19.4%)	
≥10th percentile	80 (80.0%)	55 (79.7%)	25 (80.6%)	
**Amniotic fluid volume (MVP)**				
≤3 cm	14 (14.0%)	2 (2.9%)	12 (38.7%)	<0.001
≥4 cm	86 (86.0%)	67 (97.1%)	19 (61.3%)	
**Placental location**				
Posterior wall	49 (49.0%)	43 (62.3%)	6 (19.4%)	<0.001
Anterior wall	51 (51.0%)	26 (37.7%)	25 (80.6%)	
**Tocolysis used**	65 (65.0%)	45 (65.2%)	20 (64.5%)	1.00

## Data Availability

The original contributions presented in the study are included in the article, further inquiries can be directed to the corresponding author.

## References

[1] Dahl C.M., Zhang Y.B., Ong J.X.B., Yeh C., Son M.M., Miller E.S., Roy A., Grobman W.A.M. (2021). A Multivariable Predictive Model for Success of External Cephalic Version. Obstet. Gynecol..

[2] Manasar-Dyrbus M., Seifert B., Drosdzol-Cop A., Stojko R., Staniczek J. (2025). Transforming clinical practice in just one year: Lessons from external cephalic version success. Ginekol. Pol..

[3] Manasar-Dyrbus M., Drosdzol-Cop A., Stojko S., Stojko R., Staniczek J. (2025). Strategies to reduce cesarean deliveries: Surveying Polish obstetricians on external cephalic version practices. Ginekol. Pol..

[4] Hofmeyr G.J., Kulier I.R. (2015). External cephalic version for breech presentation at term. Cochrane Database Syst. Rev..

[5] Impey L.W.M., Murphy D.J., Griffiths M., Bray E., Penna L.K. (2017). External Cephalic Version and Reducing the Incidence of Term Breech Presentation: Green-top Guideline No. 20a. BJOG Int. J. Obstet. Gynaecol..

[6] Kwiatek M., Geca T., Stupak A., Kwasniewski W., Mlak R., Kwasniewska I.A. (2024). External cephalic version—Single-center experience. Ginekol. Pol..

[7] Kok M., Van Der Steeg J.W., Mol B.W.J., Opmeer B., Van Der Post I.J.A. (2008). Which factors play a role in clinical decision-making in external cephalic version?. Acta Obstet. Gynecol. Scand..

[8] Kok M., Cnossen J., Gravendeel L., Van Der Post J.A., Mol I.B.W. (2009). Ultrasound factors to predict the outcome of external cephalic version: A meta-analysis. Ultrasound Obstet. Gynecol..

[9] Goetzinger K.R., Harper L.M., Tuuli M.G., Macones G.A., Colditz G.A. (2011). Effect of Regional Anesthesia on the Success Rate of External Cephalic Version: A Systematic Review and Meta-Analysis. Obstet. Gynecol..

[10] Kok M., Cnossen J., Gravendeel L., Van Der Post J., Opmeer B., Mol I.B.W. (2008). Clinical factors to predict the outcome of external cephalic version: A metaanalysis. Am. J. Obstet. Gynecol..

[11] Kishkovich T.P., Naert M.N., Warsame F.B., Taboada M.P., James K.E., Barth W.H.J., Clapp M.A. (2023). External Validation of a Prediction Model for External Cephalic Version Success. Obstet. Gynecol..

[12] De Hundt M., Vlemmix F., Kok M., Van Der Steeg J.W., Bais J.M., Mol B.W., Van Der Post J.A. (2012). External Validation of a Prediction Model for Successful External Cephalic Version. Am. J. Perinatol..

[13] Yerrabelli R.S., Palsgaard P.K., Shankarappa P., Jennings I.V. (2025). The Optimal Prediction Model for Successful External Cephalic Version. Am. J. Perinatol..

[14] Burgos J., Cobos P., Rodriguez L., Pijoán J.I., Fernández-Llebrez L., Martínez-Astorquiza T., Melchor J.C. (2012). Clinical score for the outcome of external cephalic version: A two-phase prospective study. Aust. N. Z. J. Obstet. Gynaecol..

[15] Tasnim N., Mahmud G., Javaid I.K. (2012). GNK-PIMS Score: A Predictive Model for Success of External Cephalic Version. J. South Asian Fed. Obstet. Gynaecol..

[16] Manasar-Dyrbuś M., Janik A., Jendyk C., Drosdzol-Cop A., Brzęk A., Stojko R., Staniczek J. (2025). Strategies to reduce cesarean deliveries: Surveying Polish midwives and midwifery students on external cephalic version practices. BMC Nurs..

[17] Collins G.S., Reitsma J.B., Altman D.G., Moons I.K. (2015). Transparent reporting of a multivariable prediction model for individual prognosis or diagnosis (TRIPOD): The TRIPOD Statement. BMC Med..

[18] Tan J.M., Macario A., Carvalho B., Druzin M.L., El-Sayed I.Y.Y. (2010). Cost-effectiveness of external cephalic version for term breech presentation. BMC Pregnancy Childbirth.

